# Quantum Statistical Complexity Measure as a Signaling of Correlation Transitions

**DOI:** 10.3390/e24081161

**Published:** 2022-08-19

**Authors:** André T. Cesário, Diego L. B. Ferreira, Tiago Debarba, Fernando Iemini, Thiago O. Maciel, Reinaldo O. Vianna

**Affiliations:** 1 Departamento de Física, ICEx, Universidade Federal de Minas Gerais (UFMG), Av. Pres. Antônio Carlos 6627, Belo Horizonte 31270-901, Brazil; 2 Departamento Acadêmico de Ciências da Natureza, Universidade Tecnológica Federal do Paraná (UTFPR), Campus Cornélio Procópio, Avenida Alberto Carazzai 1640, Cornélio Procópio 86300-000, Brazil; 3 Instituto de Física, Universidade Federal Fluminense (UFF), Niterói 24210-346, Brazil; 4 Instituto de Física, Federal University of Rio de Janeiro (UFRJ), Rio de Janeiro 21941-972, Brazil

**Keywords:** statistical complexity measure, quantum statistical complexity measure, quantum phase transitions, 1*D*-Quantum Ising Model, Heisenberg XXZ spin-1/2 Model

## Abstract

We introduce a quantum version for the statistical complexity measure, in the context of quantum information theory, and use it as a signaling function of quantum order–disorder transitions. We discuss the possibility for such transitions to characterize interesting physical phenomena, as quantum phase transitions, or abrupt variations in correlation distributions. We apply our measure on two exactly solvable Hamiltonian models: the 1D-Quantum Ising Model (in the single-particle reduced state), and on Heisenberg XXZ spin-1/2 chain (in the two-particle reduced state). We analyze its behavior across quantum phase transitions for finite system sizes, as well as in the thermodynamic limit by using Bethe Ansatz technique.

## 1. Introduction

Let us consider a physical, chemical, or biological process such as, for example, the change in the temperature of the water, the mixture between two solutions, or the formation of a neural network. It is intuitive to believe that if one can classify all the possible configurations of such systems, described by their ordering and disordering patterns, it would be possible to characterize and control them. As, for example, during the process of changing the temperature of water, by knowing the pattern of ordering and disordering of its molecular structure, it would be possible to characterize and completely control its phase transitions.

In information theory, the ability to identify certain patterns of order and disorder of probability distributions enables us to control the creation, transmission, and measurement of information. In this way, the characterization and quantification of complexity contained in physical systems and their constituent parts is a crucial goal for information theory [1]. One point of consensus in the literature about complexity is that no formal definition of this term exists. Intuition suggests that systems which can be described as “not complex” are readily comprehended: they can be described concisely by means of few parameters or variables, and their information content is low. Complexity quantifiers must satisfy some properties: (*i.*) assign a minimum value (possibly zero) for opposite extremes of order and disorder; (*ii.*) should be sensitive to transitions of order–disorder patterns; (*iii.*) and must be computable. There are considerable ways to define measures of the degree of complexity of physical systems. Among such definitions, we can mention measures based on data compression algorithms of finite size sequences [2,3,4], Kolmogorov or Chaitin measures based on the size of the smallest algorithm that can reproduce a particular type of pattern [5,6], and measures concerning the classical information theory [7,8,9,10,11,12,13]. Based on recent progress in defining a general canonical divergence within Information Geometry, a canonical divergence measure was presented with the objective of quantify complexity for both classical and quantum systems. In the classical realm, it was proven that this divergence coincides with the classical Kullback–Leibler divergence, and in the quantum domain it reduces to the quantum relative entropy [14,15].

The statistical complexity assigns the simplicity of a probability distribution to the amount of resources needed to store information [16,17]. Similarly, in the quantum realm, the complexity of a given density matrix could be translated as the resource needed to create, operate or measure the quantum state of the system [18,19,20]. On the other hand, the quantum information meaning of complexity could play an important role in the quantification of transitions of order and disorder patterns, which could indicate some quantum physical phenomenon, such as quantum phase transitions.

Regarding the complexity contained in systems, some of the simplest models in physics are the ideal gas and the perfect crystal. In an ideal gas model, the system can be found with the same probability in any of the available micro-states, therefore each state contributes equally to the same amount of information. On the other hand, in a perfect crystal, the symmetry rules restrict the accessible states of the system to only one very symmetric state. These simple models are extreme cases of minimum complexity, in a scale of order and disorder, therefore, there might exist some intermediate state which contains a maximum complexity value in that scale [21].

The main goal of this work is to introduce a quantum version of the statistical complexity measure, based on the physical meaning of the characterization and quantification of transitions between order–disorder patterns of quantum systems. As a by-product, this measure could be applied in the study of quantum phase transitions. Physical properties of systems across a quantum phase transition are dramatically altered, and in this way, it is interesting to understand how the complexity of a system would behave under such transitions. In our analysis, we studied the single particle reduced state of the parametric 1D-Ising Model and the two-particle reduced state of Heisenberg XXZ spin-1/2 Model.

The manuscript is organized as follows: In Section 2, we introduce the Statistical Measure of Complexity defined by Lópes-Ruiz et al. in [21], and in Section 3, we introduce a quantum counterpart of this measure: the Quantum Statistical Complexity Measure. We present some properties of this measure (in Section 4), and also exhibit a closed expression of this measure for one-qubit, written in the Bloch basis (Section 4.1). We also discuss two interesting examples and applications: the 1D-Quantum Ising Model (Section 4.2), in which we compute the Quantum Statistical Complexity Measure for one-qubit reduced state from *N* spins, in the thermodynamic limit, with the objective of determining the quantum phase transition point. We further determine the first-order quantum transition point and the continuous quantum phase transition for the Heisenberg XXZ spin-1/2 model, with h=0 (Section 4.3), by means of the Quantum Statistical Complexity Measure of the two-qubit reduced state for nearest neighbors, and in the thermodynamic limit. Finally, we give concluding remarks in Section 5.

## 2. Classical Statistical Complexity Measure—CSCM

Consider a system possessing *N* accessible states $\left\{x_{1},x_{2},\cdots ,x_{N}\right\}$, when observed on a particular scale, with each state having an intrinsic probability given by $\vec{p}={\left\{p_{i}\right\}}_{i=1}^{N}$. As discussed before, the candidate function to quantify the complexity of a probability distribution associated with a physical system, must attribute zero value for systems with the maximum degree of order, that is, for pure distributions: $\vec{p}=\left\{p_{i}=1,p_{j\neq i}=0\right\}$, and also assign zero for disordered systems which are characterized by an independent and identically distributed vector (i.i.d.): $\vec{I}=\left\{I_{i}=1/N\right\}$, for all i=1,…,N. Let us address the case of ordered (Section 2.1) and disordered (Section 2.2) systems separately.

### 2.1. Degree of Order

A physical system possessing the maximum degree of order can be regarded as a system with a symmetry of all of its elements. The probability distribution that describes such systems is best represented by a pure vector, which places the system as having only one possible configuration. Physically, this is the case for a gas at zero Kelvin temperature, or a perfect crystal where the symmetry rules restrict the accessible state to a very symmetric one. In order to quantify the degree of order of a given system, the function must assign maximum value for pure probability distributions, and attribute zero for equiprobable configurations. A function capable of quantifying such a degree of order is the $l_{1}$-distance between the probability distribution and the independent and identically distributed vector (i.i.d.):

(1) $$D\left(\vec{p},\vec{I}\right)=\frac{1}{2}\left|\right|\vec{p}-\vec{I}{\left|\right|}_{1}=\frac{1}{2}\sum_{i}\sqrt{{\left(p_{i}-\frac{1}{N}\right)}^{2}},$$

where $\vec{I}$, is the (i.i.d.) vector, i.e., it is a vector with elements: $I_{i}=1/N$, for all i=1,…,N. This function plays the role of the *disequilibrium* function and it quantifies the degree of order of a probability vector. It consists of the sum of the absolute values of the elements of the vector $\vec{p}-\vec{I}$. The disequilibrium measure *D* will have zero value for maximally disordered systems and maximum value for maximally ordered systems.

### 2.2. Degree of Disorder

In contrast with an ordered system, a system possessing the maximum degree of disorder is described by an equiprobable distribution. This means an equally probable expectation of occurring any of its configurations, as in a fair dice game, or in a partition function of an isolated ideal gas. From a statistical point of view, the probability vector that describes this feature is the independent and identically distributed vector (i.i.d.) $\vec{I}$, as defined above. One can define the degree of disorder of a system as a function which assigns value of zero for pure probabilities distributions, (associated with maximally ordered distributions), and a maximum value for the i.i.d. distribution. A well known function capable of quantifying the degree of disorder of a probability vector is the Shannon entropy:

(2) $$H\left(\vec{p}\right)=-\sum_{i=1}^{N}p_{i}\log\left(p_{i}\right).$$

In this way, the Shannon entropy $H(\vec{p})$ will assign zero for maximally ordered systems, and a maximal value for i.i.d vectors equals to logN. The log function is usually taken in basis 2, in order to quantify the amount of disorder in bits.

### 2.3. Quantifying Classical Complexity

Using the maximally ordered and maximally disordered states and Equations (1) and (2), respectively, Lópes-Ruiz et al. in [21] defined a classical statistical measure of complexity constructed as a product of such order–disorder quantifiers. There are intermediate states of order–disorder that may exhibit some interesting physical properties and which can be associated with complex behavior. Therefore, in this sense, this measure should deal with these intermediate states by measuring the amount of complexity of a physical system [21].

**Definition** **1**  (Classical Statistical Complexity Measure—(CSCM) [21])**.** *Let us consider a probability vector given by $\vec{p}={\left\{p_{i}\right\}}_{i=1}^{N}$, with $\dim(\vec{p})=N$, associated with a random variable X, representing all possible states of a system. The function $C(\vec{p})$ is a measure of the system’s complexity and can be defined as:*

(3) $$C\left(\vec{p}\right)=\frac{1}{\log N}H\left(\vec{p}\right)D\left(\vec{p},\vec{I}\right).$$

*The function $C(\vec{p})$ will vanish for “simple systems”, such as the ideal gas model or a crystal, and it should reach a maximum value for some state.*

**Definition** **2**  (Classical Statistical Complexity Measure of Joint Probability Distributions and of Marginal Probability Distributions)**.** *Given a known joint distribution of two discrete random variables X and Y, given by: $p(x_{i},y_{j})$, of dimension N, one can define the CSCM of the joint distribution $C_{XY}\left({\vec{p}}_{XY}\left(x_{i},y_{j}\right)\right)$, by Equation (4):*

(4) $$C_{XY}\left({\vec{p}}_{XY}\left(x_{i},y_{j}\right)\right)=\frac{1}{\log N}H\left({\vec{p}}_{XY}\left(x_{i},y_{j}\right)\right)D\left({\vec{p}}_{XY}\left(x_{i},y_{j}\right),\vec{I}\right),$$

*and in the same way, we define the CSCM of the two marginal distributions, $C_{X}\left({\vec{p}}_{X}\left(x_{i}\right)\right)$, by Equation (5) and $C_{Y}\left({\vec{p}}_{Y}\left(y_{j}\right)\right)$, by Equation (6):*

(5) $$\begin{array}{cc} & C_{X}\left({\vec{p}}_{X}\left(x_{i}\right)\right)=\frac{1}{\log N}H\left({\vec{p}}_{X}\left(x_{i}\right)\right)D\left({\vec{p}}_{X}\left(x_{i}\right),\vec{I}\right), \end{array}$$

(6) $$\begin{array}{cc} & C_{Y}\left({\vec{p}}_{Y}\left(y_{j}\right)\right)=\frac{1}{\log N}H\left({\vec{p}}_{Y}\left(y_{j}\right)\right)D\left({\vec{p}}_{Y}\left(y_{j}\right),\vec{I}\right), \end{array}$$

*where ${\vec{p}}_{X}$ and ${\vec{p}}_{Y}$ are marginal probability density functions given by: ${\vec{p}}_{X}\left(x_{i}\right)=\sum_{j=1}^{N}{\vec{p}}_{XY}\left(x_{i},y_{j}\right)$, and ${\vec{p}}_{Y}\left(y_{j}\right)=\sum_{i=1}^{N}{\vec{p}}_{XY}\left(x_{i},y_{j}\right)$.*

Generalizations of these measures defined above to continuous variables are straightforward (cf. Refs. [22,23]). In the same way, we can also generalize Definition 2 in order to deal with conditional probability densities like $\vec{p}\left(x|y\right)$ or $\vec{p}\left(y|x\right)$, which may be useful in some other more general contexts.

Classical Statistical Complexity Measure depends on the nature of the description associated to a system and with the scale of observation [24]. This function, generalized as a functional of a probability distribution, has a relation with a time series generated by a classical dynamical system [24]. Two ingredients are fundamental in order to define such a quantity: the first one is an entropy function which quantifies the information contained in a system, and could also be the Tsallis’ Entropy [25], Escort-Tsallis [26], or Rényi Entropy [27]. The other ingredient is the definition of a distance function in the state of probabilities, which indicates the disequilibrium relative to a fixed distribution (in this case the distance to the i.i.d. vector). For this purpose we can use an Euclidean Distance (or some other *p*-norm), the Bhattacharyya Distance [28], or Wootters’ Distance [29]. We can also apply a statistical measure of divergence, for example the Classical Relative Entropy [30], Hellinger distance, and also Jensen–Shannon Divergence [31]. We make note of some other generalized versions of complexity measures in recent years, and these functions have proven to be useful in some branches of classical information theory [7,32,33,34,35,36,37,38,39,40].

## 3. Quantum Statistical Complexity Measure—QSCM

### 3.1. Quantifying Quantum Complexity

The quantum version of the statistical complexity measure quantifies the amount of complexity contained in a quantum system, in an order–disorder scale. For quantum systems, the probability distribution is replaced by a density matrix (positive semi-definite and trace one). Likewise, the classical case, the extreme cases of order and disorder, are, respectively, the pure quantum states $\left|\psi \rangle \langle \psi \right|$, and the maximally mixed state: I:=I/N, where *N* is the dimension of the Hilbert space. Additionally, in analogy with the description for classical probability distributions, the quantifier of quantum statistical complexity must be zero for maximum degree of order and disorder. One can define the Quantum Statistical Complexity Measure (QSCM) as a product of an order and a disorder quantifiers: a quantum entropy, which measures the amount of disorder related to a quantum system, and a pairwise distinguishability measure of quantum states, which plays the role of a *disequilibrium* function. One of the functions to measure the amount of disorder of a quantum system is the von Neumann entropy and it is given by:

(7) S(ρ)=−Tr[ρlog(ρ)],

where ρ is the density matrix of the system. The trace distance between ρ and the the maximally mixed state quantifies the degree of order:

(8) $$D\left(\rho ,I\right)\equiv {∥\rho -I∥}_{1}=\frac{1}{2}\mathrm{Tr}\sqrt{{\left(\rho -I\right)}^{2}}.$$

For our purposes here, we define the Quantum Statistical Complexity Measure–(QSCM) in Definition 4 by means of the trace distance between the reduced quantum state ρ and I acting as the reference state. However, the trace distance function was chosen in both Definition 3 and in Definition 4 because it is the most distinguishable distance in the Hilbert space, and also monotonic under stochastic operations.

**Definition** **3**  (Quantum Statistical Complexity Measure—(QSCM))**.** *Let $\rho \in D(H_{N})$ be a quantum state over an N-dimensional Hilbert space. Then we can define the Quantum Statistical Complexity Measure as the following functional of ρ:*

(9) $$C\left(\rho \right)=\frac{1}{\log N}S\left(\rho \right)\cdot D\left(\rho ,I\right),$$

*where S(ρ) is the von Neumann entropy, and D(ρ,I) is a distinguishability quantity between the state ρ and the normalized maximally mixed state I, defined in the suitable space.*

**Definition** **4**  (Quantum Statistical Complexity Measure of the Reduced Density Matrix)**.** *Let $\rho_{SE}\in D\left(H_{NM}\right)$ be a global system of dimension NM, or any compound state of a system and its environment, and let $\rho =\rho_{S}={\mathrm{Tr}}_{E}\left[\rho_{SE}\right]\in D\left(H_{N}\right)$, (having dimension N), be the reduced state of this compound state (where ${\mathrm{Tr}}_{E}$ is the partial trace over the environment). Then we can define the Quantum Statistical Complexity Measure of the Reduced Density Matrix $C(\rho_{S})$ as:*

(10) $$\begin{array}{cc} & C\left(\rho_{S}\right)=C\left({\mathrm{Tr}}_{E}\left[\rho_{SE}\right]\right)=\frac{1}{\log N}S\left({\mathrm{Tr}}_{E}\left[\rho_{SE}\right]\right)\cdot D\left({\mathrm{Tr}}_{E}\left[\rho_{SE}\right],I\right),\\ & C\left(\rho_{S}\right)=\frac{1}{\log N}S\left(\rho_{S}\right)\cdot D\left(\rho_{S},I\right), \end{array}$$

*where $S(\rho_{S})$ is the von Neumann entropy of the quantum reduced state: $\rho_{S}={\mathrm{Tr}}_{E}\left[\rho_{SE}\right]$, and $D(\rho_{S},I)$ is a distinguishability quantity between the quantum reduced state ($\rho_{S}$), and the normalized maximally mixed state I, defined in the suitable space.*

In this work, we will use the Definition 4 (Quantum Statistical Complexity Measure of the Reduced Density Matrix) as the Quantum Statistical Complexity Measure–(QSCM). The reason for this choice is that we will study quantum phase transitions, therefore, we will apply the QSCM in the one-qubit state (reduced by *N* parts) in the thermodynamic limit, in the case of the 1D-Ising Model, (Section 4.2), and in the case of the Heisenberg Model XXZ-1/2, (Section 4.3), we will apply the QSCM in the two-qubit state (reduced of *N* parts), also in the thermodynamic limit. Definition 4 is the quantum analogue of Definition 2, i.e., it is the quantum correspondent of CSCM defined by means of “marginal probability distributions”. Extensions of these measures defined above to continuous variables are trivial.

In analogy with the classical counterpart, in the definition of quantum statistical complexity measure, there is a *carte blanche* in choosing the quantum entropy function, such as the quantum Rényi entropy [41], or quantum Tsallis entropy [42], among many others functions. Similarly, we can choose other disequilibrium functions as a measure of distinguishability of quantum states. It can be some Shatten-*p* norm [43], or a quantum Rényi relative entropy [44], the quantum skew divergence [45], or a quantum relative entropy [46]. Another feature that might generalize the quantities defined in Definition 3 and Definition 4 is to define a more general quantum state $\rho^{*}$ as a reference state (rather than the normalized maximally mixed state I) in the disequilibrium function. This choice must be guided by some physical symmetry or interest. Some obvious candidates are the thermal mixed quantum state, and the canonical thermal pure quantum state [47].

### 3.2. Some Properties of the QSCM

To complete our introduction of the quantifier of quantum statistical complexity, we should require some properties to guarantee a *bona fide* information quantifier. The amount of order–disorder, as measured by the QSCM, must be invariant under local unitary operations because it is related to the purity of the quantum reduced states.

**Proposition** **1**  (Local Unitary Invariance)**.** *The Quantum Statistical Complexity Measure is invariant under local unitary transformations, applied on the quantum reduced state of system-environment: Let a quantum system: $\rho =\rho_{S}\in D\left(H_{S}\right)$ be the quantum reduced state of dimension N, and let $\rho_{SE}\in D\left(H_{SE}\right)$ be the compound system-environment state, having dimension NM, such that: $\rho_{S}={\mathrm{Tr}}_{E}\left[\rho_{SE}\right]$, therefore:*

(11) $$C\left(U_{S}\;\rho \;U_{S}^{†}\right)=C\left(\rho \right),$$

*where $\rho =\rho_{S}\in D\left(H_{S}\right)$, and $U_{S}$ is a local unitary transformation acting on $D(H_{S})$, and ${\mathrm{Tr}}_{E}$ is the partial trace over the environment E. The extension of this property to the global state $\rho_{SE}$ is trivial.*

This statement comes directly from the invariance under local unitary transformation of von Neumann entropy and trace distance applied on the quantum reduced state $\rho_{S}$.

Another important property regards the case of inserting copies of the system in some experimental contexts. Let us consider an experiment in which the experimentalist must quantify the QSCM of a given state ρ by means of a certain number *n* of copies $\rho^{⊗n}$, which therefore implies that the QSCM of the copies should be bounded by the quantity of only one copy.

**Proposition** **2**  (Sub-additivity over copies). *Given a product state $\rho^{⊗n}$, with dim(ρ)=N, the QSCM is a sub-additive function over copies:*

(12) $$C\left(\rho^{⊗n}\right)\leq nC\left(\rho \right).$$

Indeed this is an expected property for a measure of information, since the regularized number of bits of information gained from a given system cannot increase just by considering more copies of the same system. The proof of Proposition 2 is in Appendix A, and it comes from the additivity of von Neumann entropy and sub-additivity of trace distance.

It is important to notice that quantum statistical complexity is not sub-additive over general extensions with quantum states, for example: *i.* Extensions with maximally mixed states: let us consider a given state ρ is extended with one maximally mixed state I=I/N, with dim(ρ)=dim(I)=N.

(13) $$\begin{array}{cc} C(\rho ⊗I)\; & =\frac{S(\rho )+\log N}{2\log N}D\left(\rho ,I\right), \end{array}$$

(14) $$\begin{array}{cc} \; & =\frac{C(\rho )}{2}+\frac{D(\rho ,I)}{2}, \end{array}$$

(15) $$\begin{array}{cc} \; & \leq \frac{C(\rho )}{2}+\frac{1}{2}. \end{array}$$

Equation (15) presents an upper bound to the QSCM for this extended state. This feature demonstrates that the measure of the compound state is bounded by the quantity of one copy. *ii.* In Equation (18) we present the QSCM for a *more general extension given by* $\rho^{⊗n}⊗I^{⊗n}$. This feature shows that the measure of the compound state is also bounded by the quantity of one copy.

(16) $$\begin{array}{cc} C(\rho^{⊗n}⊗I^{⊗n}) & \leq n\frac{S(\rho )+\log N}{2\log N}D\left(\rho ,I\right), \end{array}$$

(17) $$\begin{array}{cc} \; & \leq n\left(\frac{C(\rho )}{2}+\frac{D(\rho ,I)}{2}\right), \end{array}$$

(18) $$\begin{array}{cc} \; & \leq n\left(\frac{C(\rho )}{2}+\frac{1}{2}\right). \end{array}$$

*iii.* As a last example of *nonextensivity over general compound states*, let us consider the extension with a pure state $\left|\psi \rangle \langle \psi \right|$, with $\dim\left(\rho \right)=\dim\left(\left|\psi \rangle \langle \psi \right|\right)$.

(19) $$\begin{array}{cc} C(\rho ⊗\left|\psi \rangle \langle \psi \right|) & =\frac{S(\rho )}{2\log N}D\left(\rho ,\left|\psi \rangle \langle \psi \right|\right), \end{array}$$

(20) $$\begin{array}{cc} \; & \leq \frac{S(\rho )}{2\log N}\left(D\left(\rho ,I\right)+\frac{N-1}{N}\right). \end{array}$$

As discussed above, the QSCM is a measure that intends to detect changes in properties, as for example changes on patterns of order and disorder. Therefore, the measure must be a continuous function over the parameters of the states responsible for its transitional characteristics. Naturally, the quantum complexity is a continuous function, since it comes from the product of two continuous functions. Due to continuity, it is possible to define the derivative function of the quantum statistical complexity measure:

**Definition** **5**  (Derivative)**.** *Let us consider a physical system described by the one-parameter set of states: $\rho \left(\alpha \right)\in D\left(H_{N}\right)$, for α∈R. We can define the derivative with respect to α as:*

(21) $$\frac{dC}{d\alpha }:=\lim_{\varepsilon \to 0}\frac{C(\rho (\alpha +\varepsilon ))-C(\rho (\alpha ))}{\varepsilon }.$$

In the same way as defined in Definition 5, it is possible to obtain higher order derivatives.

**Definition** **6**  (Correlation Transition)**.** *In many-particle systems a transition of correlations occurs when a system changes from a state that has a certain order, pattern or correlation, to another state possessing another order pattern or correlation.*

At low temperatures, physical systems are typically ordered, increasing the temperature of the system, they can undergo phase transitions or order–disorder transitions into less ordered states: solids lose their crystalline form in a solid–liquid transition; iron loses magnetic order if heated above the Curie point in a ferromagnetic-paramagnetic transition, etc. The description of physical systems depends on measurable quantities such as temperature, interaction strength, interaction range, orientation of an external field, etc. These quantities can be described by parameters in a suitable space. For example, let us consider a parameter describing some physical quantity α, and a set of one parameter state ρ(α).

Phase transitions are characterized by a sharp change in the complexity of the physical system that exhibits such emergent phenomena when this suitable control parameter α exceeds a certain critical value. The study of transitions between correlations with the objective of inferring physical properties of a system can generate great interest. We know that at the phase transition point, the reduced state of *N* particles undergoes abrupt transitions that go through states that have a certain purity and change abruptly to reduced mixed states. These transitions can indicate a certain type of correlation transition that can be detected. For many-particle and composed systems, quick change on the local order–disorder degree can be associated with a transition in the correlations pattern. In this way, a detectable change in these parameters may indicate an alteration in system configuration, which is considered here as a change in the pattern of order–disorder.

Quantum phase transition is a fundamental phenomenon in condensed matter physics, and is tightly related to quantum correlations. Quantum critical points for Hamiltonian models with external magnetic fields at finite temperatures were studied extensively. In the Quantum Information scenario, the quantum correlation functions used in these studies of quantum phase transitions concerned almost only concurrence and quantum discord. The behavior of quantum correlations for the Heisenberg XXZ spin-1/2 chain via negativity, information deficit, trace distance discord, and local quantum uncertainty was investigated in [48]. However, other measures of quantum correlations had also been proposed in order to detect quantum phase transitions, such as: local quantum uncertainty [49], entanglement of formation [50,51], quantum discord, and classical correlations [52]. Authors in Ref. [53] revealed a quantum phase transition in an infinite 1D-XXZ chain by using concurrence and Bell inequalities. The behaviors of the quantum discord, quantum coherence, and Wigner–Yansase skew information and the relations between the phase transitions and symmetry points in the Heisenberg XXZ spin-1 chains have been broadly investigated in Ref. [54]. The ground state properties of the one-dimensional extended Hubbard model at half filling from the perspective of its particle reduced density matrix were studied in [55], where the authors focused on the reduced density matrix of two fermions and performed an analysis of its quantum correlations and coherence along the different phases of the model.

In an abstract manner, a quantum state undergoing a path through the i.i.d. identity matrix is an example of such transitions which may have physical meaning, as we will observe later in some examples. Let us suppose that a certain subspace of a quantum system can be interpreted as having a certain order (i.e., a degree of purity of the compound state of *N* particles), and there exists a path in which this subspace passes through the identity. This path can be analyzed as having an order–disorder transition. In order to illustrate the formalism of quantum statistical complexity in this context of order–disorder transition, in Section 4 we apply it to two well-known quantum systems that exhibit quantum phase transitions: the 1D-Quantum Ising Model (Section 4.2), and the Heisenberg XXZ spin-1/2 model (Section 4.3).

## 4. Examples and Applications

In this section we calculate an analytic expression for the Quantum Statistical Complexity Measure (QSCM), of one-qubit, written in the Bloch basis, (Section 4.1), and present the application of QSCM in order to evince quantum phase transitions and correlation ordering transitions for the 1D-Quantum Ising Model (Section 4.2) and for the Heisenberg XXZ spin-1/2 model (Section 4.3).

### 4.1. QSCM of One-Qubit

Let us suppose we have a one-qubit state ρ, written in the Bloch basis. In Equation (22), we analytically exhibit the Quantum Statistical Complexity Measure C(ρ) of one-qubit, written as: $\rho =\frac{1}{2}\left(I+\vec{r}\cdot \vec{\sigma }\right)$:

(22) $$C\left(r\right)=-\frac{r^{2}}{2}\mathrm{arctanh}\left(r\right)-\frac{r}{4}\log\left(\frac{1-r^{2}}{4}\right).$$

where $\vec{r}=\left(x,y,z\right)$, and $r=|\vec{r}|=\sqrt{x^{2}+y^{2}+z^{2}}$, with 0≤r≤1, and $\vec{\sigma }$ is the Pauli matrix vector. Normalization constants such as log(2), for example, are omitted in Equation (22) just for aesthetic reasons.

It is interesting to notice that the Quantum Statistical Complexity Measure of one-qubit, written in the Bloch basis, C(ρ) is a function dependent only on *r*, that is, C(r). This expression will be useful in the study of quantum phase transitions, for example, in the 1D-Ising Model, discussed in Section 4.2, where an analytical expression for the state of one-qubit reduced from *N* spins, in the thermodynamic limit, will be obtained. Other useful expressions can be obtained, for example, the trace distance between the state and the normalized identity for one-qubit is also a function of *r*, in the Bloch’s basis, D(r)=r/2, and therefore, the entropy function can be easily written as S(r)=2C(r)/r by using Equation (22). In addition, we exhibit analytic expressions for the first (Equation (23)), and the second, Equation (24)) derivatives of QSCM, for one-qubit, written in the Bloch Basis. One can observe that these functions also depend only on *r*:

(23) $$\frac{dC(r)}{dr}=-r\cdot \mathrm{arctanh}\left(r\right)-\frac{1}{4}\log\left(\frac{1-r^{2}}{4}\right),$$

(24) $$\frac{d^{2}C\left(r\right)}{dr^{2}}=-\frac{r}{2\left(1-r^{2}\right)}-\mathrm{arctanh}\left(r\right).$$

### 4.2. 1D Quantum Ising Model

The 1D-Quantum Ising Model presents a quantum phase transition and, despite its simplicity, still generates a lot of interest from the research community. One of the motivations lies in the fact that spin chains possess a great importance in modelling quantum computers. The Hamiltonian of the 1D-Quantum Ising Model is given by:

(25) $$H=-J\sum_{j=1}^{N}\sigma_{j}^{x}\sigma_{j+1}^{x}-g\sigma_{j}^{z},$$

where $\left\{\sigma^{x},\sigma^{y},\sigma^{z}\right\}$ are the Pauli matrices, *J* is an exchange constant that sets the interaction strength between the pairs of first neighbors {j,j+1}, and *g* is a parameter that represents an external transverse field. Without loss of generality, we can set J=1, since it simply defines an energy scale for the Hamiltonian. The Ising model ground state can be obtained analytically by a diagonalization consisting of three steps:

- A Jordan–Wigner transformation:
  $$\sigma_{j}^{z}⟶1-2c_{j}^{†}c_{j};$$
  where $c_{j}$ and $c_{j}^{†}$ are the annihilation-creation operators, respecting the anti-commutation relations: $\left\{c_{j},c_{k}^{†}\right\}=\delta_{jk}I$, and $\{c_{j},c_{k}\}=0$;
- A Discrete Fourier Transform (DFT):
  $$c_{j}⟶\frac{1}{\sqrt{N}}\sum_{k=0}^{N-1}c_{k}\;e^{2\pi i(kj)/N};$$
- A Bogoliubov transformation:
  $$c_{k}⟶\cos\left(\theta_{k}/2\right)\gamma_{k}-\sin\left(\theta_{k}/2\right)\gamma_{-k}^{†};$$
  where $\theta_{k}$ represents the basis rotation from the mode $c_{k}$ to the new mode representation $\gamma_{k}$. The angles $\theta_{k}$ are chosen such that the ground state of the Hamiltonian in Equation (25) is the vacuum state in $\gamma_{k}$ mode representation, and it is given by $\theta_{k}=\arctan\left(\frac{\sin(k)}{g-\cos(k)}\right)$ 56].

We can calculate the reduced density matrix of one spin by using the Bloch representation, in which all coefficients are obtained via expectation values of Pauli operators. The one-qubit state in the site *j*, given by $\rho_{j}^{\left(1\right)}$ can be written as:

(26) $$\rho_{j}^{\left(1\right)}=\frac{I}{2}+\frac{{\vec{r}}_{j}\cdot {\vec{\sigma }}_{j}}{2},$$

where $r_{j}^{a}=\langle \sigma_{j}^{a}\rangle$ are expected values in vacuum state in the site *j*, and a=x,y,z. Note that $\langle \sigma_{j}^{x}\rangle =\langle \sigma_{j}^{y}\rangle =0$, ∀j, because they combine an odd number of fermions. Therefore, the Bloch’s vector possesses only the *z*-component. Let us define $\theta_{k}/2=\beta_{k}$, and $\theta_{k^{\prime }}/2=\beta_{k^{\prime }}$. Thus, the *z*-component will be given by:

$$\begin{array}{cc} \sigma_{j}^{z} & =1-2c_{j}^{†}c_{j},\\ \sigma_{j}^{z} & =1-\frac{2}{N}\sum_{k,k^{\prime }}e^{-i(k-k^{\prime })j}c_{k}^{†}c_{k^{\prime }},\\ \sigma_{j}^{z} & =1-\frac{2}{N}\sum_{k,k^{\prime }}e^{-i(k-k^{\prime })j}\left(\cos\left(\beta_{k}\right)\gamma_{k}^{†}-\sin\left(\beta_{k}\right)\gamma_{-k}\right)\left(\cos\left(\beta_{k^{\prime }}\right)\gamma_{k^{\prime }}-\sin\left(\beta_{k^{\prime }}\right)\gamma_{-k^{\prime }}^{†}\right).\\ \sigma_{j}^{z} & =1-\frac{2}{N}\sum_{k,k^{\prime }}e^{-i(k-k^{\prime })j}\left(\cos\left(\beta_{k}\right)\cos\left(\beta_{k^{\prime }}\right)\gamma_{k}^{†}\gamma_{k^{\prime }}-\right.\left.\cos\left(\beta_{k}\right)\sin\left(\beta_{k^{\prime }}\right)\gamma_{k}^{†}\gamma_{-k^{\prime }}^{†}\right.\\ & \left.-\sin\left(\beta_{k}\right)\cos\left(\beta_{k^{\prime }}\right)\gamma_{-k}\gamma_{k^{\prime }}\right.\left.+\sin\left(\beta_{k}\right)\sin\left(\beta_{k^{\prime }}\right)\gamma_{-k}\gamma_{-k^{\prime }}^{†}\right). \end{array}$$

As discussed above, the only non-vanishing term will be $\langle \sigma_{j}^{z}\rangle$, and therefore:

(27) $$\begin{array}{cc} \langle \sigma_{j}^{z}\rangle & =1-\frac{2}{N}\sum_{k,k^{\prime }}e^{-i(k-k^{\prime })j}\left(\cos\left(\beta_{k}\right)\cos\left(\beta_{k^{\prime }}\right)\langle \gamma_{k}^{†}\gamma_{k^{\prime }}\rangle -\right.\left.\cos\left(\beta_{k}\right)\sin\left(\beta_{k^{\prime }}\right)\langle \gamma_{k}^{†}\gamma_{-k^{\prime }}^{†}\rangle \right.\\ & \left.-\sin\left(\beta_{k}\right)\cos\left(\beta_{k^{\prime }}\right)\langle \gamma_{-k}\gamma_{k^{\prime }}\rangle \right.\left.+\sin\left(\beta_{k}\right)\sin\left(\beta_{k^{\prime }}\right)\langle \gamma_{-k}\gamma_{-k^{\prime }}^{†}\rangle \right),\\ \langle \sigma_{j}^{z}\rangle & =1-\frac{2}{N}\sum_{k,k^{\prime }}e^{-i(k-k^{\prime })j}\sin\left(\beta_{k}\right)\sin\left(\beta_{k^{\prime }}\right)\delta_{k,k^{\prime }},\\ \langle \sigma_{j}^{z}\rangle & =1-\frac{2}{N}\sum_{k}\sin^{2}\left(\theta_{k}/2\right). \end{array}$$

In Equations (28) we exhibit the one-qubit reduced density matrix in the Bloch basis ($\rho_{1}$):

(28) $$\rho_{1}=\frac{I}{2}+\left(\frac{1}{2}-\frac{1}{N}\sum_{k\in K}\sin^{2}\left(\frac{\theta_{k}}{2}\right)\right)\sigma^{z},$$

where the angle $\theta_{k}$ is the Bogoliubov rotation angle and the summation index k∈K, with $K=[\pm \frac{\pi }{N},\pm \frac{3\pi }{N},\cdots ,\pm \left(\pi -\frac{2\pi }{N}\right)]$. This result is independent of the spin index, as expected for systems that are translational invariant.

We can now calculate QSCM for the reduced density matrix analytically. From Equation (22), we simply identify the Bloch vector of the reduced density matrix having only *z*-component, as written in Equation (27). This quantity in the thermodynamic limit can be obtained by taking the limit of the Riemann sums, $\langle \sigma^{z}\rangle \left(g\right)=\lim_{N\to \infty }\sum_{k=1}^{N}\langle \sigma_{k}^{z}\rangle$. This Bloch’s vector component is a function of the field *g*, that is, $\langle \sigma^{z}\rangle \left(g\right)=\lim_{N\to \infty }\sum_{k=1}^{N}1-\frac{2}{N}\sum_{k}\sin^{2}\left(\theta \left(k\right)\right)$, with $\theta \left(k\right)=\arctan\left(\frac{\sin(l)}{g-\cos(l)}\right)$, and $l=\frac{(2k-1)\pi }{N}-\pi$. Thus, the *z* component of Bloch’s vector given in Equation (27) goes to the following integral, written as:

(29) $$\langle \sigma^{z}\rangle \left(g\right)=1-\frac{2}{\pi }\int_{-\pi }^{0}\sin^{2}\left(\frac{1}{2}\arctan\left(\frac{\sin(\xi )}{g-\cos(\xi )}\right)\right)d\xi .$$

This integral can be solved analytically in the thermodynamic limit for some values of the transverse field parameter. For g=0, we can easily obtain $\langle \sigma_{z}\rangle =0$, which corresponds to a one-qubit maximally mixed reduced state $\rho_{1}=I/2$. At g=1, i.e., in the critical point, the integral given in Equation (29) can be also solved and we obtain $\langle \sigma_{z}\rangle =2/\pi$, in the thermodynamic limit. The eigenvalues of the one-qubit reduced state $\rho_{1}$, at g=1, can be obtained analytically as: {1/2±1/π}. For other values of *g*, the integral written in Equation (29) can be written as elliptic integrals of first and second kinds [57]. By using the result given in Equation (29) on Equation (22), we can thus obtain the quantum statistical complexity measure for one-qubit reduced density matrix in the thermodynamic limit as a function of the transverse field parameter *g*.

**Figure 1:**
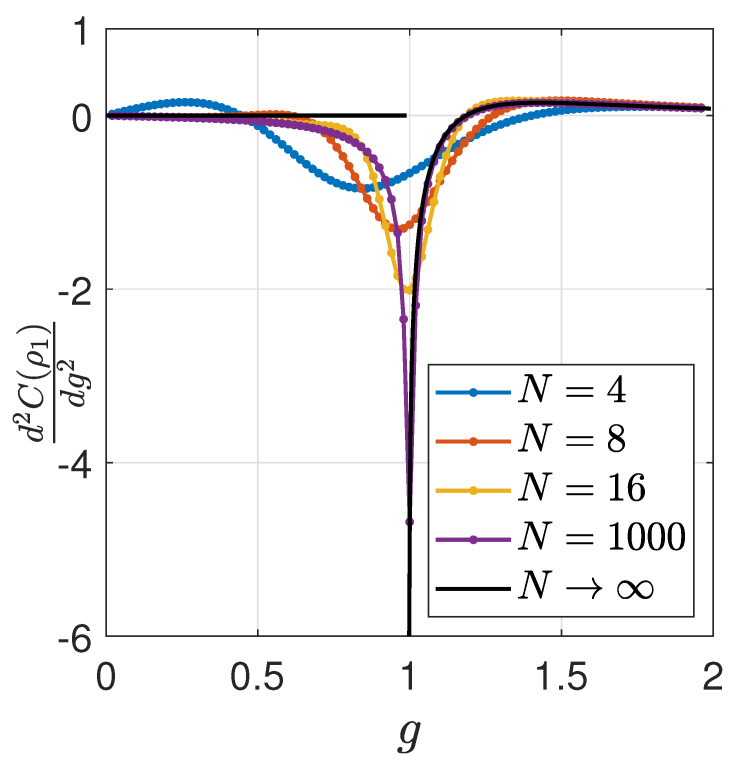
Second derivative of QSCM as a function of *g*. The Second derivative of QSCM with respect to the transverse field parameter *g*, for different finite system sizes: N=4,6,16,1000, and for the thermodynamic limit (continuous line), N→∞, for g∈[0,2].

In Figure 1 we present the second derivative of QSCM with respect to the transverse field parameter *g*, for different finite system sizes N=4,8,16,1000. We also calculated this derivative in the thermodynamic limit by using Equation (24) and Equation (29).

It is well known that at g=1 there is a quantum phase transition of second order [58]. By observing Figure 1, we can directly recognize a sharp behavior of the measure in the transition point.

### 4.3. XXZ-½ Model

Quantum spin models as the XXZ-1/2 can be simulated experimentally by using Rydberg-excited atomic ensembles in magnetic microtrap arrays [59], and also by a low-temperature scanning tunneling microscopy [60], among many other quantum simulation experimental arrangements. Let us consider a Heisenberg XXZ spin-1/2 model defined by the following Hamiltonian:

$$H=-J\sum_{j=1}^{N}\left[S_{j}^{x}S_{j+1}^{x}+S_{j}^{y}S_{j+1}^{y}+\Delta S_{j}^{z}S_{j+1}^{z}\right]-2h\sum_{j=1}^{N}S_{j}^{z},$$

with periodic boundary conditions, $S_{j+N}^{\alpha }=S_{j}^{\alpha }$, and $S_{j}^{\alpha }=\frac{1}{2}\sigma_{j}^{\alpha }$, where $\sigma_{j}^{\alpha }$ are Pauli matrices, and Δ is the uni-axial parameter strength, which is a ratio of $S^{z}$ interactions between $S^{x}$ or $S^{y}$ interactions. This model can interpolate continuously between classical Ising, quantum XXX, and quantum XY models. At Δ=0, it turns to the quantum XY or XX0 model which corresponds to free fermions on a lattice. For Δ=1, (Δ=−1), the anisotropic XXZ model Hamiltonian reduces to the isotropic (ferro)anti-ferromagnetic XXX model Hamiltonian. For Δ→±∞, the model goes to an (ferro)anti-ferromagnetic Ising Model.

The parameter *J* defines an energy scale and only its sign is important: we observe a ferromagnetic ordering along the x−y plane for positive values of *J*, and, for negative ones, we notice the anti-ferromagnetic alignment. The uni-axial parameter strength Δ distinguishes a planar regime x−y (when |Δ|<1), from the axial alignment, (for |Δ|>1), cf. [61]. Thereby, it is useful to define two regimes: for |Δ|>1, the *Ising-like* regime and |Δ|<1, the *XY-like regime* in order to model materials possessing respectively an easy-axis and easy-plane magnetic anisotropies [62].

Here we are interested in quantum correlations between the nearest and next to nearest neighbor spins in the XXZ spin-1/2 chain with J=1, at a temperature of 0K, and zero external field (h=0). The matrix elements of $\varrho_{i+r}$ are written in function of expectation values which mean the correlation functions for nearest neighbor r=1, (for $\varrho_{i+1}$), and the correlation functions for next-to-nearest neighbors r=2, (for $\varrho_{i+2}$), and they are given by a set of integral equations which can be found in Appendix B or in [48,53,63,64,65]. These two point correlation functions for the XXZ model at zero temperature and in the thermodynamic limit can be derived by using the Bethe Ansatz technique. In Equation (30), due to the symmetry in the Hamiltonian model, it is presented the two-qubit reduced density matrix of sites *i* and i+r, for r=1,2, in the thermodynamic limit, written in the basis |1〉=|↑↑〉, |2〉=|↑↓〉, |3〉=|↓↑〉 and |4〉=|↓↓〉, where |↑〉 and |↓〉 are the eigenstates of the Pauli *z*-operator [48]:

(30) $$\varrho_{i+r}=\left(\begin{array}{cccc} \varrho_{11} & 0 & 0 & 0\\ 0 & \varrho_{22} & \varrho_{23} & 0\\ 0 & \varrho_{32} & \varrho_{33} & 0\\ 0 & 0 & 0 & \varrho_{44} \end{array}\right),$$

where $\varrho_{11}=\frac{1+\langle \sigma_{i}^{z}\sigma_{i+r}^{z}\rangle }{4}$, $\varrho_{23}=\frac{\langle \sigma_{i}^{x}\sigma_{i+r}^{x}\rangle }{2}$, and $\varrho_{22}=\frac{1-\langle \sigma_{i}^{z}\sigma_{i+r}^{z}\rangle }{4}$, with $\varrho_{11}=\varrho_{44}$, $\varrho_{23}=\varrho_{32}$ and $\varrho_{22}=\varrho_{33}$.

**Figure 2:**
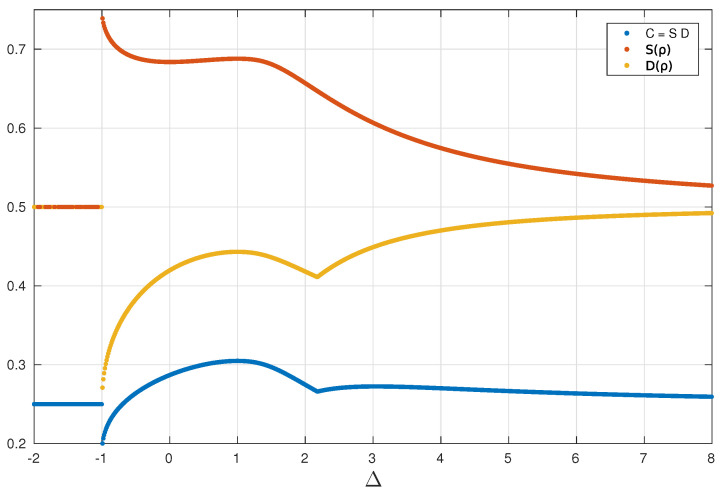
Comparison between measures. Quantum Statistical Complexity Measure $C(\varrho_{i+1})$ (blue), von Neumann Entropy $S(\varrho_{i+1})$ (orange), and the disequilibrium function given by the Trace distance $D(\varrho_{i+1},I)$ (yellow) in function of Δ. All measures were calculated for the two-qubit reduced density matrix of sites *i* and i+1, $\varrho_{i+1}$, for Δ∈[−1,8], in the thermodynamic limit.

In Figure 2 we show the QSCM, $C(\varrho_{i+1})$, for nearest neighbor, in contrast with the von Neumann entropy $S(\varrho_{i+1})$ and the trace distance $D(\varrho_{i+1},I)$ between ϱ and the normalized identity matrix, all as a function of the uni-axial parameter strength Δ. The XXZ model possess two critical points: the first-order transition occurs at Δ=−1, and also a continuous phase transition shows up at Δ=1 [66]. An interesting feature of the QSCM is the fact that it evinces points of correlation transitions, related to the order–disorder transitions, which may not necessarily be connected with phase transitions. In this respect, we take note of the cusp point in Figure 2, at Δ≈2.178.

In order to investigate the cusp point of $C(\varrho_{i+1})$ at Δ=2.178, let us consider what happens with the state $\varrho_{i+1}$, given by Equation (30), as Δ varies. The state $\varrho_{i+1}$ given in Equation (30) can be easily diagonalized, thus let us study the following matrix $\varrho_{i+1}-I/4$, which plays an important role in the quantum statistical complexity measure as already discussed. This matrix has the following eigenvalues: $\{\frac{1}{4}\left(2\langle \sigma_{i}^{x}\sigma_{i+1}^{x}\rangle -\langle \sigma_{i}^{z}\sigma_{i+1}^{z}\rangle \right),\frac{1}{4}(-2\langle \sigma_{i}^{x}\sigma_{i+1}^{x}\rangle$$-\langle \sigma_{i}^{z}\sigma_{i+1}^{z}\rangle ),\frac{1}{4}\langle \sigma_{i}^{z}\sigma_{i+1}^{z}\rangle ,\frac{1}{4}\langle \sigma_{i}^{z}\sigma_{i+1}^{z}\rangle \}$. As the Δ value increases in the interval [1,3], correlation values in the *x* direction also increase while correlations in *z* decrease, reaching the local minimum observed in Figure 2. In this interval, the eigenvalue $2\langle \sigma_{i}^{x}\sigma_{i+1}^{x}\rangle -\langle \sigma_{i}^{z}\sigma_{i+1}^{z}\rangle$ goes through zero, and this, therefore, should cause the correlation transition. This correlation transition is due to the fact that this eigenvalue vanishes for some Δ in this interval, which should imply a change of orientation of spin correlations.

By following this reasoning, in order to determine such points at which changes of orientation of spin correlations occur, it is necessary to solve numerically some integral equations, given by the eigenvalues of Equation (30), which are functions of expected values given in Refs. [48,53,63,64,65]. This procedure has the objective of determining the solution for which values of Δ the following integral equation holds: $2\langle \sigma_{i}^{x}\sigma_{i+1}^{x}\rangle -\langle \sigma_{i}^{z}\sigma_{i+1}^{z}\rangle =0$. Due to the fact that the values of $\langle \sigma_{i}^{y}\sigma_{i+1}^{y}\rangle =\langle \sigma_{i}^{x}\sigma_{i+1}^{x}\rangle$, for this Hamiltonian, the solution of this equation indicates the point where planar xy-correlation decreases while *z*-correlation increases, although we are already in the ferromagnetic phase. For Δ→∞, the system moves towards a configuration that exhibits correlation only in the *z*-direction. Proceeding in the same way, by solving the other integral equation given in the eigenvalues set: $-2\langle \sigma_{i}^{x}\sigma_{i+1}^{x}\rangle -\langle \sigma_{i}^{z}\sigma_{i+1}^{z}\rangle =0$, we obtain a divergence solution for which Δ=−1.

**Figure 3:**
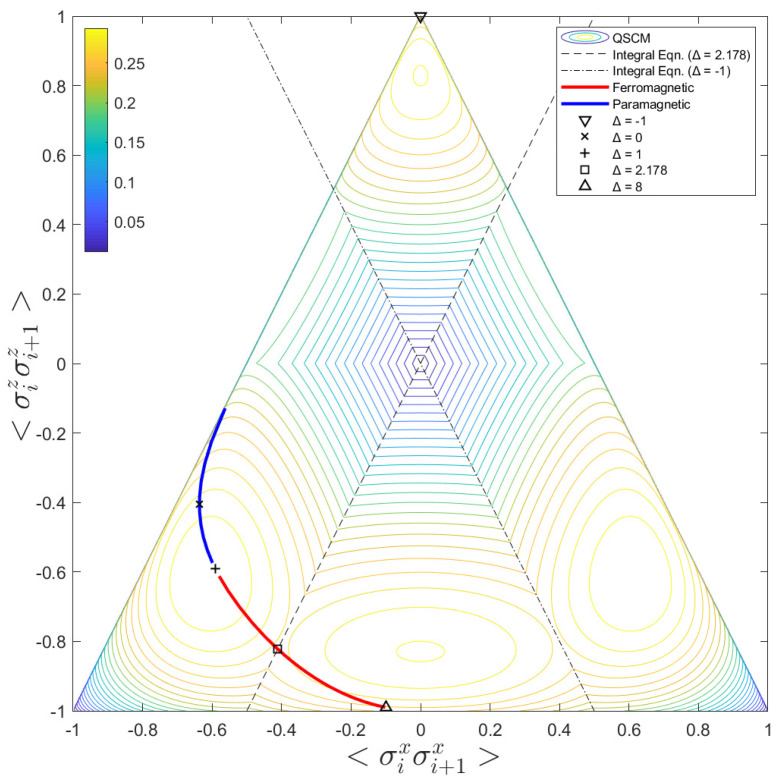
Contour map. The contour map of QSCM, $C(\varrho_{i+1})$, in function of the correlation functions $\langle \sigma_{i}^{x}\sigma_{i+1}^{x}\rangle$ and $\langle \sigma_{i}^{z}\sigma_{i+1}^{z}\rangle$. The dash inclined straight line represents the integral equation whose solution is Δ=2.178, and the dash-point straight line represents the curve for Δ=−1, for which there is a divergence point. The indicated path inside the contour map shows the curve performed by the variation of $C(\varrho_{i+1})$, inside the positive semi-definite density matrix space, for Δ∈[−1,8]. The highlighted points are: Δ=−1, (∇); Δ=0, (×); Δ=1, (+); Δ=2.178, (☐) and Δ=8, (Δ). Additionally, the ferromagnetic region (red) and the paramagnetic region (blue) are also represented in this path.

In Figure 3, we call attention to a contour map of $C(\varrho_{i+1})$ in function of $\langle \sigma_{i}^{x}\sigma_{i+1}^{x}\rangle$, and $\langle \sigma_{i}^{z}\sigma_{i+1}^{z}\rangle$. The triangle region represents the convex hull of positive semi-definite density matrices. The vertices of this triangle are given by: $\left(\langle \sigma_{i}^{x}\sigma_{i+1}^{x}\rangle ,\langle \sigma_{i}^{z}\sigma_{i+1}^{z}\rangle \right)=\{\left(-1,-1\right);\;$(0,1); and (1,−1)}. Along with the contour map of QSCM as a function of the correlation functions in *x* and *z* directions, the integral equations obtained while the two eigenvalues of $\varrho_{i+1}-I/4$ goes to zero are also represented in Figure 3. These integral equations are represented by the two inclined straight lines (the dash and dash-dot ones). The dash and inclined straight line describes the integral equation whose solution is Δ=2.178. The dash-dot straight line represents the curve for Δ=−1 solution, for which there exists a divergence point (the phase transition point).

As previously mentioned, QSCM showed to be sensitive to correlation transitions. In Figure 3, the thick and colorful curve inside the contour map shows the path taken by $C(\varrho_{i+1})$, while the values of correlations in *x* and *z* vary when Δ increases monotonically in the interval [−1,8]. This same path was also presented in Figure 2, on the blue curve. The blue part of the thick curve represents values for the correlations in which we have a paramagnetic state, and the red part of the thick and colorful curve indicates the values for the ferromagnetic arrangement. Additionally, we have highlighted some interesting points in this colorful curve by a ×: for Δ=−1, (∇); for Δ=0, (×); Δ=1, (+); Δ=2.178, (☐) and for Δ=8, (Δ).

**Figure 4:**
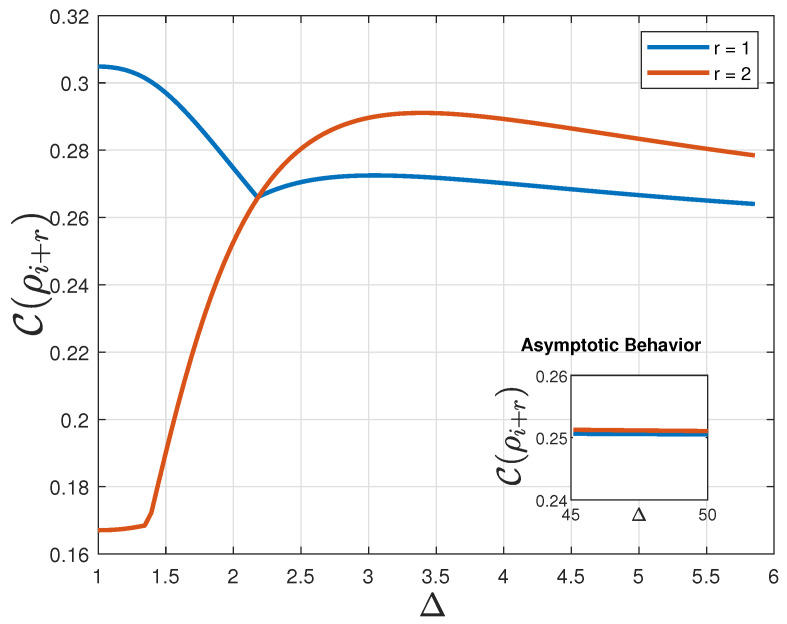
QSCM for nearest neighbors, (r=1), and for next-to-nearest neighbors (r=2). $C(\varrho_{i+r})$ for the two-qubit reduced density matrix of sites *i* and i+r, for r=1 (blue), and for r=2 (orange), both in the thermodynamic limit in function of the uni-axial parameter strength Δ. (**a**) Sub-Figure: Asymptotic Behavior. The sub-figure shows the asymptotic behavior for large Δ for both cases (in fact, both $C(\varrho_{i+1})$ and $C(\varrho_{i+2})\to 1/4$, when Δ→∞).

Figure 4 shows QSCM for nearest neighbors, given by $C(\varrho_{i+1})$, (blue), and for next-to-nearest neighbors, written as $C(\varrho_{i+2})$, (orange), both in the thermodynamic limit in function of the uni-axial parameter strength Δ.

The asymptotic limit for both measures (r=1,2) is also presented in the sub-figure. As Δ→∞, the behavior of these two correlation functions $\langle \sigma_{i}^{x}\sigma_{i+1}^{x}\rangle \to 0$ and $\langle \sigma_{i}^{z}\sigma_{i+1}^{z}\rangle \to -1$, for both cases. In this limit, the density matrix of the system can be written as $\varrho_{i+r}\to diag\left\{0,1/2,1/2,0\right\}$, for both cases and thus, $S(\varrho_{i+r})\to 1/2$. Additionally, $D(\varrho_{i+r},I)\to 1/2$, which makes $C(\varrho_{i+r})\to 1/4$, for r=1 and for r=2. It is interesting to notice that $C\left(\varrho_{i+1}\right)=C\left(\varrho_{i+2}\right)$ exactly at Δ=2.178. The QSCM for nearest neighbor is greater than the QSCM for next-to-nearest neighbors, i.e.,;$C\left(\varrho_{i+1}\right)>C\left(\varrho_{i+2}\right)$, for −1≤Δ<2.178. For Δ>2.178, $C\left(\varrho_{i+1}\right)<C\left(\varrho_{i+2}\right)$, until both goes to 1/4, for large values of Δ. This behavior can be an indication of a probable increase in complexity as the transition of order–disorder occurs. Therefore, it should be expected that this measure could act as a complexity pointer. Considering more correlations possible if we count on the action of second neighbors, it is understandable that an increase of complexity should occur at Δ=2.178.

## 5. Conclusions

We introduced a quantum version for the statistical complexity measure, the Quantum Statistical Complexity Measure (QSCM), and displayed some of its properties. The measure has demonstrated to be useful and physically meaningful. It possesses several of the expected properties for a *bona fide* complexity measure and demonstrates its possible usefulness in other areas of quantum information theory.

We presented two applications of the QSCM, investigating the physics of two exactly solvable quantum Hamiltonian models, namely: the 1D-Quantum Ising Model and the Heisenberg XXZ spin-1/2 chain, both in the thermodynamic limit. Firstly we calculated the QSCM for one-qubit, in the Bloch’s base, and we determined this measure as a function of the magnitude of the Bloch vector *r*. We computed its magnitude in the thermodynamic limit, first by analytically calculating the measure for the one-qubit state reduced density matrix from *N* spins. Later, in order to study the quantum phase transition for the 1D-Quantum Ising Model, we performed the limit N→∞. For the 1D-Quantum Ising Model, we obtained the quantum phase transition point at g=1. In this way, we have found that the QSCM can be used as a signaling of quantum phase transitions for this model.

Secondly, we studied the Heisenberg XXZ spin-1/2 chain, and by means of QSCM we evince a point at which a correlation transition occurs for this model. Physically, at Δ=2.178, the planar xy-correlation decreases while the *z*-correlation increases, reaching a minimum point, although we are already in the ferromagnetic arrangement. This competition between these two different alignments of correlations indicates an order–disorder transition in which the measure was shown to be sensitive.

We have studied the derivatives of the QSCM and they demonstrated to be sensitive to the quantum transition points. As a summary of this study for the Heisenberg XXZ spin-1/2, we can list: (*i*) the Quantum Statistical Complexity Measure, characterizing the first-order quantum phase transition at Δ=−1, (*ii*) and evinces the continuous quantum phase transition at Δ=1, and (*iii*) witnesses order–disorder transition at Δ=2.178, related to the alignment of the spin correlations.

## Data Availability

Not applicable.

## Abbreviations

The following abbreviations are used in this manuscript:

CSCM	Classical Statistical Complexity Measure
QSCM	Quantum Statistical Complexity Measure

## Appendix A. Sub-Additivity over Copies

**Proposition** **A1.** 
*Given a product state $\rho^{⊗n}\in D\left(H^{⊗n}\right)$, the QSCM is a sub-additive function:*

(A1)
$$C\left(\rho^{⊗n}\right)\leq nC\left(\rho \right).$$

*Equality holds if we choose the disequilibrium function to be the quantum relative entropy, S(ρ||I)=logN−S(ρ), which is additive under tensor products. In this case, the measure will be additive: $C\left(\rho^{⊗n}\right)=nC\left(\rho \right)$.*

**Proof.** Von Neumann entropy is additive for product states: $S\left(\rho^{⊗n}\right)=nS\left(\rho \right)$. This means that information contained in an uncorrelated system $\rho^{⊗n}$, is equal to the sum of its constituents parts. This equality also holds for Shannon Entropy. However, the trace distance is sub-additive with respect to the tensor product: $D\left(\rho ⊗\rho^{\prime },I⊗I\right)\leq D\left(\rho ,I\right)+D\left(\rho^{\prime },I\right),\;\forall \;\rho ,\rho^{\prime }$ [67]. We will prove this proposition by induction and also we will only consider states with same dimension, i.e.,$\dim\left(\rho^{⊗n}\right)=\dim\left(I^{⊗n}\right),\forall \;n$. It is easy to observe that the proposition is true for n=1. Let us suppose now, as an induction step, that for some arbitrary n=k>1,k∈N, $C\left(\rho^{⊗k}\right)\leq kC\left(\rho \right)$.

(A2) $$\begin{array}{cc} C(\rho^{⊗k+1}) & =\frac{S(\rho^{⊗k}⊗\rho )}{\log(N^{k+1})}D\left(\rho^{⊗k}⊗\rho ,I^{⊗k+1}\right), \end{array}$$

(A3) $$\begin{array}{cc} C(\rho^{⊗k+1}) & =\left(k+1\right)\frac{S(\rho )}{\log(N^{k+1})}D\left(\rho^{⊗k}⊗\rho ,I^{⊗k+1}\right), \end{array}$$

(A4) $$\begin{array}{cc} C(\rho^{⊗k+1}) & =\frac{S(\rho )}{\log(N)}D\left(\rho^{⊗k}⊗\rho ,I^{⊗k+1}\right). \end{array}$$

Using the sub-additivity property for the trace distance: $D\left(\rho^{⊗k},I\right)\leq kD\left(\rho ,I\right)$, and also using $S\left(\rho^{⊗k}\right)=kS\left(\rho \right)$, for an arbitrary n=k+1

(A5) $$\begin{array}{cc} C(\rho^{⊗k+1}) & \leq \left(k+1\right)\frac{S(\rho )}{\log(N)}D\left(\rho ,I\right), \end{array}$$

(A6) $$\begin{array}{cc} \; & \leq (k+1)C(\rho ). \end{array}$$

Therefore $C\left(\rho^{⊗n}\right)\leq nC\left(\rho \right),\;\;\forall \;n.$ □

## Appendix B. Correlation Functions for Nearest Neighbors and Next-to-Nearest Neighbors

The correlation functions for nearest neighbors (r=1) spins of XXZ-1/2 model. The two-point correlation functions of this model at zero temperature and in the thermodynamics limit can be derived by using the Bethe Ansatz technique [66]. The spin–spin correlation functions between nearest-neighbors spin sites for Δ>1 are given by Takahashi et al. in [63]:

$$\begin{array}{cc} & \langle \sigma_{i}^{z}\sigma_{i+1}^{z}\rangle =1+2\int_{-\infty +i/2}^{\infty +i/2}\frac{dx}{\sinh(\pi x)}\left(\cot\left(\nu x\right)\coth\left(\nu x\right)-\frac{x}{\sin^{2}\left(\nu x\right)}\right),\\ & \langle \sigma_{i}^{x}\sigma_{i+1}^{x}\rangle =\int_{-\infty +i/2}^{\infty +i/2}\frac{dx}{\sinh(\pi x)}\left(\frac{x}{\sin^{2}\left(\nu x\right)}\cosh\nu -\frac{\cot(\nu x)}{\sinh\nu }\right), \end{array}$$

with $\nu =\cosh^{-1}\Delta$. For Δ=1, we have $\langle \sigma_{i}^{x}\sigma_{i+1}^{x}\rangle =\langle \sigma_{i}^{z}\sigma_{i+1}^{z}\rangle =1/3\left(1-4\ln2\right)$, and for Δ≤−1, $\langle \sigma_{i}^{z}\sigma_{i+1}^{z}\rangle =1$ and $\langle \sigma_{i}^{x}\sigma_{i+1}^{x}\rangle =0,$ (see [53]). For −1<Δ<1, the correlation functions are given by Kato et al. in Ref. [64]:

$$\begin{array}{cc} & \langle \sigma_{i}^{z}\sigma_{i+1}^{z}\rangle =1-\frac{2}{\pi^{2}}\int_{-\infty }^{\infty }\frac{dx}{\sinh x}\frac{x\cosh x}{\cosh^{2}\left(\Phi x\right)}+\frac{2\cot(\pi \Phi )}{\pi }\int_{-\infty }^{\infty }\frac{dx}{\sinh x}\frac{\sinh((1-\Phi )x)}{\cosh(\Phi x)},\\ & \langle \sigma_{i}^{x}\sigma_{i+1}^{x}\rangle =\frac{\cos(\pi \Phi )}{\pi^{2}}\int_{-\infty }^{\infty }\frac{dx}{\sinh x}\frac{x\cosh x}{\cosh^{2}\left(\Phi x\right)}-\frac{1}{\pi \sin(\pi \Phi )}\int_{-\infty }^{\infty }\frac{dx}{\sinh x}\frac{\sinh((1-\Phi )x)}{\cosh(\Phi x)}, \end{array}$$

where $\Phi =\frac{1}{\pi }\cos^{-1}\Delta$.

The correlation functions for next-to-nearest neighbors (r=2) spins of XXZ-1/2 model. For the next-to-nearest-neighbors spins, in the region Δ>1, we have the correlation functions (see [63]) are:

$$\begin{array}{ccc} \langle \sigma_{i}^{x}\sigma_{i+2}^{x}\rangle & =\int_{-\infty +i/2}^{\infty +i/2}\frac{dx}{\sinh(\pi x)}\frac{1}{2}\left[-\frac{x}{\sin^{2}\left(\nu x\right)}\left(\frac{3\sinh^{2}\nu }{\sin^{2}\left(\nu x\right)}+1-3\cosh2\nu \right)\right.+ & \\ & +\left.\cot\left(\nu x\right)\left(\frac{3\cosh(2\nu )\tanh(\nu )}{\sin^{2}\left(\nu x\right)}-\frac{4}{\sinh(2\nu )}\right)\right], & \\ \langle \sigma_{i}^{z}\sigma_{i+2}^{z}\rangle & =1+\int_{-\infty +i/2}^{\infty +i/2}\frac{dx}{\sinh(\pi x)}\left[\frac{x}{\sin^{2}\left(\nu x\right)}\left(\frac{3\sinh^{2}\nu }{\sin^{2}\left(\nu x\right)}-1-\cosh\left(2\nu \right)\right)\right)- & \\ & \left.-\cot\left(\nu x\right)\left(\frac{3\tanh\nu }{\sin^{2}\left(\nu x\right)}-4\coth\left(2\nu \right)\right)\right], \end{array}$$

where $\nu =\cosh^{-1}\Delta$, $\Delta \leq -1,\langle \sigma_{i}^{z}\sigma_{i+1}^{z}\rangle =1$, and $\langle \sigma_{i}^{x}\sigma_{i+1}^{x}\rangle =0$. For −1<Δ<1, we have (see [65,68]):

$$\begin{array}{ccc} \langle \sigma_{i}^{x}\sigma_{i+2}^{x}\rangle & =-\int_{-\infty }^{\infty }\frac{dx}{\sinh x}\left[\frac{\sinh(1-\Phi )x}{\cosh(\Phi x)}\left(\frac{2}{\pi \sin(2\pi \Phi )}+\frac{3\cos2\pi \Phi \tan\pi \Phi }{\pi^{3}}x^{2}\right)+\right. & \\ & \left.+\frac{\cosh x}{{\left(\cosh\Phi x\right)}^{2}}\left(\frac{\cos2\pi \Phi }{\pi^{2}}x+\frac{{\left(\sin\pi \Phi \right)}^{2}}{\pi^{4}}x^{3}\right)\right], \end{array}$$

and, also:

$$\begin{array}{cccc} \langle \sigma_{i}^{z}\sigma_{i+2}^{z}\rangle & =1+4\int_{-\infty }^{\infty }\frac{dx}{\sinh x}\frac{\sinh(1-\Phi )x}{\cosh\Phi x}\left[\frac{\cot(2\pi \Phi )}{\pi }+\frac{3\tan\pi \Phi }{2\pi^{3}}x^{2}\right]- & & \\ & -4\int_{-\infty }^{\infty }\frac{dx}{\sinh x}\frac{\cosh x}{{\left(\cosh\Phi x\right)}^{2}}\left[\frac{x}{2\pi^{2}}+\frac{{\left(\sin\pi \Phi \right)}^{2}}{2\pi^{4}}x^{3}\right]. & \end{array}$$
